# “Hot” alkaline hydrolysis of UiO-66 to enhance quercetin loading, *in vitro* release, antioxidant, and anti-inflammatory properties

**DOI:** 10.3389/fnut.2025.1697265

**Published:** 2025-11-18

**Authors:** Ruimiao Chang, Huichun Wang, Shumian Liu, Shuai Jia, Jingjing Wang, Ying Song, Xiaolu Ji, Qingqing Li

**Affiliations:** 1 Department of Pharmacy, Shanxi Medical University, Taiyuan, Shanxi, China; 2 Third Hospital of Shanxi Medical University, Shanxi Bethune Hospital, Shanxi Academy of Medical Sciences, Taiyuan, Shanxi, China; 3 Tongji Medical College, Tongji Hospital, Huazhong University of Science and Technology, Wuhan, Hubei, China; 4 Key Laboratory of Cellular Physiology, Shanxi Medical University, Ministry of Education, Taiyuan, Shanxi, China; 5 Medicinal Basic Research Innovation Center of Chronic Kidney Disease, Shanxi Medical University, Ministry of Education, Taiyuan, Shanxi, China; 6 Department of Pharmacy, Health Science Center, Xi'an Jiaotong University, Xi'an, Shaanxi, China

**Keywords:** thermal alkaline hydrolysis, small size, adsorption capacity, quercetin, *in vitro* release

## Abstract

**Background:**

The Food and Drug Administration (FDA) classifies quercetin (QU) as a generally recognized as safe (GRAS) substance and exhibits striking antioxidant and anti-inflammatory activities. However, its extremely low solubility in water limits its application in the fields of food and nutrition. Nanomaterials such as UiO-66 (University of Oslo 66) have been widely used in the field of nutrient delivery.

**Methods:**

In this study, terephthalic acid ligands were stripped from small-sized UiO-66 via a thermal alkaline hydrolysis method, resulting in a new drug-loaded material, UiO-66-BH-100 (UiO-66 blended and heated, with a size of 100 nm). The obtained UiO-66-BH-100 showed significantly increased porosity and good thermal stability. UiO-66-BH-100, which is rich in hydroxyl groups (1.7 × 1019/m^2^), could dramatically enhance the adsorption capacity for QU (302.60 vs. 135.57 mg/g). The results of adsorption experiments indicate that the adsorption of QU by UiO-66-BH-100 tends to follow a monolayer adsorption behavior, suggesting that electron pair sharing or transfer may occur during the adsorption process.

**Results:**

The QU-loaded drug carrier UiO-66-BH-100@QU has good biocompatibility and could significantly improve the in vitro release behavior of QU. Notably, the capacity of UiO-66-BH-100@QU to scavenge diphenyl-2-picrylhydrazyl (DPPH) and 22'-azinobis (3-ethylbenzothiazoline-6-sulfonic acid) diammonium salt (ABTS), as well as its ability to inhibit NO secretion, was almost comparable to that of QU, demonstrating promising antioxidant and anti-inflammatory potential.

**Conclusion:**

This study provides a simple and effective new approach for delivering natural active ingredients with excellent bioavailability in the field of functional foods.

## Graphical Abstract

**Figure:**
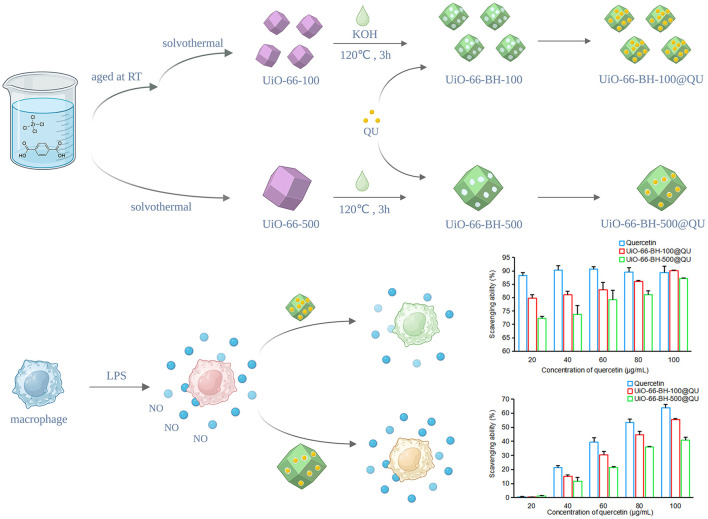

## 1 Introduction

Quercetin (QU), a natural flavone compound, is ubiquitously present in different fruits and vegetables, for example, apples, onions, and broccoli (1, 2), as well as in tea and propolis (3). Extensive studies have demonstrated that QU exhibits remarkable antioxidant (4), anti-inflammatory (5), and antibacterial activities (6). Recognized for its safety, QU has been designated as “Generally Recognized as Safe” by the U.S. FDA (7). In the field of the food industry, QU has been widely used as a natural preservative, nutritional enhancer, and functional food ingredient (8). Based on this solid market application foundation, the clinical application prospects of QU in disease prevention and treatment are becoming increasingly broad. Notably, the unique planar molecular structure of QU results in tight molecular packing and strong intermolecular forces, rendering it poorly dispersible in solvents. Consequently, QU exhibits extremely low solubility in water (9). Additionally, its stability is highly susceptible to environmental factors, including oxygen concentration, pH, temperature, metal ions, and the presence of antioxidants. These characteristics lead to suboptimal stability within the gastrointestinal tract, a short biological half-life, and low oral bioavailability following oral administration (10, 11). This greatly limits the potential application of QU in the clinical treatment of diseases.

During the last few years, metal–organic framework (MOF) materials have demonstrated unique advantages in the field of drug delivery due to their high drug loading capacity and controlled release characteristics (12, 13). As a typical Zr-based MOF, UiO-66 exhibits excellent chemical stability and biocompatibility (14) and has a wide range of applications in the release of bioactive ingredients (15–17). The pore size of UiO-66 (~0.8 nm) highly matches the molecular size of QU (0.4 × 0.7 nm) (18). Loading QU into UiO-66 is expected to effectively improve the solubility and release behavior of QU, thereby enhancing its bioavailability. However, the original UiO-66 does not perform ideally when loading QU (18, 19), and it is necessary to modify UiO-66 using appropriate methods.

Interestingly, the ligand terephthalic acid in UiO-66 material has a group similar to QU. If the ligand in UiO-66 was removed, it might greatly increase the loading capacity of UiO-66 for QU. Thermal alkaline hydrolysis technology is a simple, inexpensive, fast, and effective method for removing MOF ligands. It uses high-temperature alkaline hydrolysis technology to strip ligands from MOFs, thereby obtaining a new type of material. Thermal alkaline hydrolysis technology could change the composition, crystal structure, density, surface structure, adsorption properties, etc., of MOF materials (20), demonstrating great potential in regulating the properties of MOF materials. Previous literature has shown that materials obtained through alkaline hydrolysis have better stability than MOF precursors and a larger specific surface area (21). Based on this, we speculate that the use of thermal alkaline hydrolysis technology to remove organic ligands from UiO-66 might optimize the loading effect of QU by generating new holes and performance changes in the material.

Extensive research has established that drug-loaded materials with smaller particle sizes critically modulate payload capacity, release kinetics, and therapeutic efficacy (22, 23). Generally, drug-loaded materials with smaller particle sizes often exhibit an enhanced specific surface area and higher surface energy. This not only provides more adsorption sites for drugs but also enhances the interaction between the drug-loaded materials and drug molecules, thereby increasing the drug payload capacity. During the drug release stage, due to the larger surface contact area, a drug delivery system with small particle sizes could enable drugs to diffuse and release more rapidly (24, 25). Moreover, in the *in vivo* environment, drug delivery systems with small particle sizes have better dispersibility and permeability and can penetrate biological membranes more efficiently, thus improving the drug absorption efficiency (25).

At present, there are still few studies on optimizing UiO-66 by reducing the material's particle size and combining appropriate methods to improve its performance in loading and releasing QU. This study aimed to modify small-particle-size UiO-66 via thermal alkaline hydrolysis technology to obtain a new material with significantly enhanced QU loading capacity, and it is further expected to improve QU's *in vitro* release profile as well as its anti-inflammatory and antioxidant properties. We first optimized the synthesis method to prepare UiO-66 with a small particle size and treated it with the thermal alkaline hydrolysis technology. The obtained small-particle-size material may enhance its hydrogen bond interaction with QU by increasing the surface area (from 107 to 292 m^2^/g) and the surface hydroxyl density (1.7 × 10^19^/m^−2^), thereby increasing the loading capacity of QU. This study systematically investigates the effects of reducing particle size and stripping ligands on the loading, adsorption behavior, adsorption mechanism, and release behavior of UiO-66 on QU and verifies its anti-inflammatory and antioxidant properties through DPPH radical scavenging experiments, ABTS scavenging experiments, and nitric oxide (NO) release inhibition experiments. The material developed in this study could effectively improve the release behavior of QU and is expected to enhance its bioavailability in food systems, thereby enabling QU to exert its natural nutritional functions, such as antioxidant and anti-inflammatory effects, more stably and efficiently in the development of functional foods.

## 2 Materials and methods

### 2.1 Materials

QU (>98%) was procured from Macklin Biochemical Co. (China). Terephthalic acid (>99%), ZrCl4 (>98%), 22′-azinobis(3-ethylbenzothiazoline-6-sulfonic acid) diammonium salt (ABTS), and methyl thiazolyl tetrazolium bromide (MTT) were procured from Shanghai Aladdin Biochemical Co., Ltd. RAW264.7 mouse mononuclear macrophages were purchased from the Institute of Life Sciences (Shanghai, China). Lipopolysaccharide (LPS, purity >98.0%) and 1,1-diphenyl-2-picrylhydrazyl (DPPH, purity >98.0%) were purchased from Solarbio Science & Technology Co., Ltd. (Beijing, China); DMEM was purchased from Boster Biological Engineering Co., Ltd. (Wuhan, China); the Griess method NO detection kit was purchased from Beyotime Biotechnology Co., Ltd. (Shanghai, China); fetal bovine serum was purchased from EIA Industrial Co., Ltd. (Jiangsu, China); water used in the experiment was manufactured by Milli-Q element (18.2 X, Millipore, Massachusetts), and other reagents were of analytical grade.

### 2.2 Synthesis of the UiO-66

UiO-66-500 with a hydrated particle size of approximately 500 nm was synthesized according to the method reported in the literature (26, 27). To reduce the particle size of the crystals, during the synthesis of UiO-66-100, a small amount of water was added to the solvent, and the amount of acetic acid (used as a crystal growth regulator) was reduced. The specific synthesis process was as follows: 1.05 g of zirconium tetrachloride was taken and added to 30 ml of N, N-dimethylformamide (DMF). After it was dissolved and stirred evenly, 1.9 ml of water and 4.2 ml of acetic acid were added. After aging at room temperature for 1 day, 15 ml of a DMF solution containing 0.3 M terephthalic acid was added, and the reaction was conducted at 120 °C for 24 h. After the products were separated by centrifugation, they were washed 2–3 times with DMF and absolute ethanol, respectively, followed by controlled vacuum desiccation at 60 °C for 12 h to harvest UiO-66-100.

### 2.3 Thermal alkaline hydrolysis treatment

The thermal alkaline hydrolysis materials were prepared as follows: Aliquots of the material were dispersed in a 0.2 M potassium hydroxide (KOH) solution (solid–liquid ratio 0.1 g: 30 ml) at ambient temperature. The suspension was then sealed in polytetrafluoroethylene-lined autoclaves and subjected to hydrothermal treatment at 120 °C for precisely 3 h. Post-processing measures involved cooling to an indoor temperature and rinsing with deionized water and ethyl alcohol (3–5 cycles), respectively. Finally, the material was dried under vacuum at 150 °C for 3 h to obtain UiO-66-BH-100 (UiO-66-100 blended and then heated). UiO-66-RT-100 (UiO-66-100 at room temperature) was obtained by mixing UiO-66-100 with 0.2 M KOH and reacting at room temperature for 3 h. For UiO-66-SH-100 (UiO-66-100 separately heated), UiO-66-100 and 0.2 M KOH were heated to 120 °C separately and then mixed for reaction at 120 °C for 3 h.

### 2.4 Characterization of materials

The surface topography of the materials was characterized by field-emission scanning electron microscopy (FE-SEM, SU8600, Hitachi). The hydrodynamic diameter distribution was quantified via dynamic light scattering (DLS, Zetasizer Nano ZS90, Malvern). For Fourier transform infrared spectroscopy (FTIR) analysis, accurately weighed dried samples were analyzed using a Thermo Scientific Nicolet iS5. Thermogravimetric analysis (TGA) of the materials was conducted by a ZCT-B DSC/TGA calorimeter (Jingyi Gaoke Instrument Co., Ltd., China). Nitrogen adsorption–desorption analysis tests were performed using a TriStar3000 (Micromeritics, USA) within the relative pressure range of 0.05 to 1 P/Po to analyze the specific surface area and pore architecture of the materials.

### 2.5 Study on adsorption performance

#### 2.5.1 Isothermal adsorption experiment

A series of 5.0 mg aliquots of the material was precisely weighed and transferred into sterile polypropylene conical tubes (15 ml). Subsequently, 5 ml of QU solutions with a six-point concentration gradient (100, 300, 500, 700, 900, and 1,000 μg/ml) was prepared and transferred into each tube. After sealing the tubes with parafilm, they were agitated on an orbital shaker at 25 °C and 300 rpm for 120 min. Following equilibration, the suspensions were filtered, and supernatant drug concentrations were quantified by ultraviolet (UV) spectroscopy.

The equilibrium adsorption capacity was calculated using Equation 1:


$$\begin{array}{l} Q_{e}=\left(C_{o}-C_{e}\right)\times \frac{V}{W} \end{array}$$ (1)

where *Q*_e_ (mg/g) denotes mass-normalized equilibrium adsorption capacity of QU; *C*_o_ (μg/ml) denotes initial bulk phase concentration of QU; *C*_e_ (μg/ml) denotes residual concentration at solid–liquid equilibrium of QU; *V* (ml) denotes solution volume; and *W* (mg) denotes degassed adsorbent mass.

To further describe the adsorption process of QU by the material, two models, Langmuir (Equation 2) and Freundlich (Equation 3), were used to fit the isothermal adsorption data.


$$\begin{array}{l} \frac{C_{e}}{Q_{e}}=\frac{C_{e}}{Q_{m}}+\frac{1}{K_{l}\times Q_{m}} \end{array}$$ (2)

$$\begin{array}{l} \log Q_{e}=m\log C_{e}+\log K_{f} \end{array}$$ (3)

where *Q*_e_ (mg/g) denotes equilibrium adsorption capacity; *C*_e_ (mg/L) denotes mass concentration of QU in the equilibrium solution after adsorption; *K*_l_ (L/mg) denotes the Langmuir constant; *Q*_m_ denotes the maximum theoretical adsorption capacity; *m* denotes heterogeneity factor (*m* = 1/*n*); and *K*_f_ (mg/g) denotes the Freundlich constant.

#### 2.5.2 Adsorption kinetic experiment

Six portions of the material, each approximately 5.0 mg, were weighed and placed into 15-ml centrifuge tubes. Then, 5 ml of a 1,000 μg/ml QU ethanol solution was added to each tube. After sealing the tubes, they were placed in a constant-temperature shaker at 25 °C and 300 rpm. Centrifuge tubes were retrieved at specified intervals (1, 10, 30, 60, 90, and 120 min). After filtration, the supernatant was collected, and the residual QU content was quantified. The amount of QU adsorbed onto the material was calculated by determining the residual QU concentration in the supernatant.

The pseudo-first-order (PFO; Equation 4) and pseudo-second-order (PSO; Equation 5) models were applied to characterize the adsorption kinetic behavior:


$$\begin{array}{l} \ln\left(Q_{e}-Q_{t}\right)=\ln Q_{e}-k_{1}t \end{array}$$ (4)

$$\begin{array}{l} \frac{t}{Q_{t}}=\frac{1}{k_{2}\times Q_{e}\times Q_{e}}+\frac{t}{Q_{e}} \end{array}$$ (5)

where *Q*_e_ (mg/g) represents the equilibrium adsorption capacity, *Q*_t_ (mg/g) denotes the time-dependent adsorption capacity at a specific time “*t*,” and *k*_1_ and *k*_2_ correspond to the rate constants of the PFO and PSO kinetic equations, respectively.

### 2.6 Loading

Based on the experimentally calculated adsorption amount of QU by the material and referring to the method in the literature (28, 29), the material and QU were mixed in appropriate proportions, they were added separately to 1 ml of ethanol, and the mixture was stirred at room temperature in the dark until the solvent was completely volatilized, yielding a powdered product. The powdered product was washed with ultrapure water three times and then vacuum-dried at 40 °C overnight, thus obtaining the drug delivery systems: UiO-66-BH-100@QU (UiO-66-BH-100 loaded with QU) and UiO-66-BH-500@QU (UiO-66-BH-500 loaded with QU). Calculation of drug loading capacity: 2 mg of the QU-loaded material was dispersed in ethanol under continuous stirring at room temperature for 1 h to facilitate drug release. Subsequent centrifugation (10,000 rpm, 10 min) enabled supernatant collection, and the QU concentration was quantified by ultraviolet–visible (UV-Vis) spectroscopy. This extraction–centrifugation cycle was repeated until negligible QU was detected in the supernatant. The cumulative summation of QU was calculated to determine the total drug loading recovered from all supernatants. The drug loading capacity (DLC%) was determined using Equation 6:


$$\begin{array}{l} \text{DLC }\left(\text{\%}\right)=\frac{{\text{W}}_{\text{A}}}{{\text{W}}_{\text{B}}}\times 100 \end{array}$$ (6)

where *W*_A_ stands for the mass of the drug in the drug delivery system (mg), and *W*_B_ stands for the total mass of the drug delivery system and the drug (mg).

### 2.7 Release

To investigate the release behavior of quercetin in the drug-loaded system in simulated gastric fluid (SGF, pH 1.2) and simulated intestinal fluid (SIF, pH 7.4), the experiment was conducted as follows: 2 mg of the drug-loaded material was weighed and dispersed in 1 ml of 1% Tween 80 solution, which was then quickly transferred into a dialysis bag and sealed. The sealed dialysis bag was moved into a 40 ml SGF system (containing 1% Tween 80) and incubated at 37 °C with shaking at 200 rpm for 2 h. Subsequently, the dialysis bag was taken out of the SGF and transferred into a system containing 40 ml of SIF (containing 1% Tween 80), followed by incubation at 37 °C with shaking at 200 rpm for 4 h.

The sampling strategy during the entire release experiment was as follows: in the SGF incubation stage, the samples were taken every 15 min; in the SIF incubation stage, the samples were taken every 30 min. For each sampling, 1.5 ml of the release medium was collected for drug concentration determination. At the same time, an equal volume of fresh release medium at the same temperature was supplemented to maintain a constant volume of the release system. The cumulative release percentage of quercetin was calculated according to Equation 7:


$$\begin{array}{l} Q_{n}=\frac{\left(C_{n}\times V_{0}+V_{i}\sum_{i=1}^{n-1}C_{i}\right)\times 100\%}{m} \end{array}$$ (7)

where *Q*_n_ represents the cumulative release rate (%) at the n-th sampling; *C*_n_ represents the drug concentration in the release medium at the n-th sampling time (mg/ml); *C*_i_ represents the drug concentration in the release medium at the i-th sampling time (mg/ml); *V*_0_ represents the volume (ml) of the release medium; *V*_i_ represents the sampling volume (ml) at the i-th sampling; and *m* represents the total drug loading amount (mg).

Meanwhile, a control experiment was performed by dispersing an equivalent mass of free QU (equivalent to the QU content in a 2 mg drug delivery system) in phosphate-buffered saline (PBS) buffer, with release kinetics assessed using the same protocol.

### 2.8 Study on antioxidant performance

#### 2.8.1 DPPH performance

A stock solution of QU or vitamin C (Vc) was prepared in absolute ethanol and serially diluted to final concentrations of 20, 40, 60, 80, and 100 μg/ml. For the drug delivery systems, solutions were prepared such that the QU content was normalized to that of the free-drug standards. Specifically, UiO-66-BH-100@QU was dispersed in absolute ethanol to yield suspensions at 95, 190, 285, 380, and 475 μg/ml, while UiO-66-BH-500@QU was dispersed to 150, 300, 450, 600, and 750 μg/ml. All solutions were prepared fresh before use.

The DPPH assay was performed following the method described in references (30–32). Briefly, a 0.1 mM DPPH solution was prepared in absolute ethanol. Aliquots of 4 ml DPPH solution were mixed with 4 ml of each sample solution, and the mixtures were incubated in the dark for 30 min. The absorbance at 517 nm was measured using a UV-Vis spectrophotometer, and the DPPH radical scavenging activity (%) was calculated using Equation 8. All measurements were performed in triplicate.


$$\begin{array}{l} \text{DPPH Scavenging Ability }\left(\text{\%}\right)=1-\frac{{{\text{A}}_{\text{t}}-\text{A}}_{\text{b}}}{{\text{A}}_{\text{c}}}\times 100 \end{array}$$ (8)

where *A*_t_ represents the absorbance of the sample-DPPH mixture, *A*_b_ represents the absorbance of the sample with anhydrous ethanol, and *A*_c_ represents the absorbance of ethanol-DPPH mixture.

#### 2.8.2 ABTS performance

Following the methods described in references (30–32), a 7.4 mM ABTS solution and a 2.6 mM potassium persulfate solution were prepared using ultrapure water. The two solutions were mixed in equal volumes and reacted at 25 °C in the dark for 16 h to obtain the stock solution that generates ABTS free radicals. An appropriate amount of the ABTS stock solution was diluted with phosphate-buffered saline (PBS) to an absorbance of 0.70 ± 0.02 at 734 nm, yielding the ABTS working solution. Subsequently, 40 μl of the sample solution was mixed with 4 ml of the ABTS working solution and reacted for 5 min. With PBS as the blank control, the absorbance was measured at 734 nm, and the ABTS scavenging capacity of the sample was determined according to Equation 9. All experiments were performed in triplicate.


$$\begin{array}{l} \text{ABTS Scavenging Ability }\left(\text{\%}\right)=\frac{{{\text{A}}_{\text{c}}-\text{A}}_{\text{s}}}{{\text{A}}_{\text{c}}}\times 100 \end{array}$$ (9)

where *A*_c_ is the absorbance of an equal volume of absolute ethanol after reacting with the ABTS working solution, and *A*_s_ is the absorbance of the sample solution after reacting with the ABTS working solution.

### 2.9 Cytotoxicity

RAW264.7 cells in the logarithmic growth phase were taken, and the cells were treated with the materials at concentrations of 5, 10, 15, and 20 μg/ml, respectively. The cell viability (*CV*) was calculated using Equation 10:


$$\begin{array}{l} CV=\frac{A_{t}}{A_{c}}\times 100\% \end{array}$$ (10)

where *CV* represents cell viability, *A*_t_ is the absorbance value (a.u.) corresponding to the cell lysate after treatment with the material, and *A*_c_ is the absorbance value (a.u.) corresponding to the cell lysate of the control group.

### 2.10 Inhibitory effect on NO

RAW264.7 cells in the logarithmic growth phase were taken and cultured until they reached a resting state. The cells were grouped and treated as follows: In the standard control and the LPS-challenged group, DMEM culture medium was added; in the drug-treated groups, DMEM culture medium containing QU or the drug delivery system was added, with the final concentrations of QU being 2.5, 5, and 10 μg/ml, respectively, and the cells were cultured for 4 h. Subsequently, the culture medium was discarded. DMEM culture medium was added to the standard control group; DMEM culture medium containing 1 μg/ml LPS was added to the LPS-challenged group and each drug-treated group, and the culture was continued. At 6, 12, 18, and 24 h of culture time, respectively, the cell culture supernatants were collected, and the content of NO in the supernatants of each group was determined using a Griess kit.

### 2.11 Statistical analysis

Three independent experimental replicates were performed under standardized conditions. Quantitative data were expressed as mean ± SD and underwent normality verification before parametric analysis. Significant intergroup differences (*p* < 0.05) were determined using one-way ANOVA with Tukey–Kramer *post-hoc* testing using SPSS software.

## 3 Results and discussion

### 3.1 Adsorption experiments

#### 3.1.1 Isothermal adsorption experiment

To elucidate the adsorption dynamics and underlying mechanisms of QU uptake, isothermal adsorption studies were performed, aiming to correlate material-specific saturation capacities with dissolved analyte concentrations.

**Figure 1:**
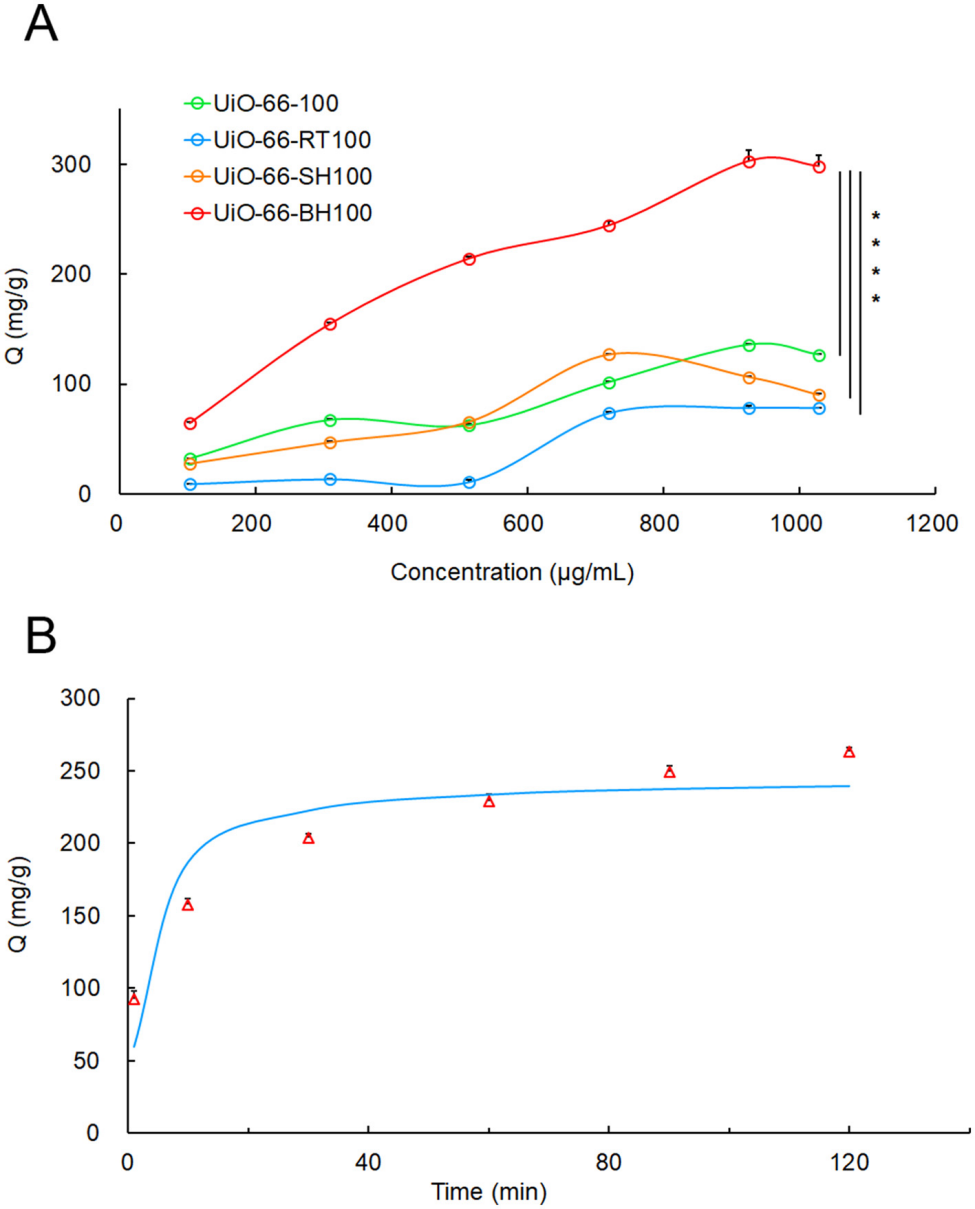
The adsorption isotherms of QU at different concentrations on UiO-66-BH-100 **(A)**, and the adsorption kinetics of UiO-66-BH-100 for QU, **(B)** the data points in the figure represent measured values, and the curve was fitted with a PSO kinetic model), *n* = 3. Statistical significance difference against UiO-66-BH-100 (****p < 0.0001).

As shown in Figure 1A, UiO-66-BH-100 exhibited the most outstanding adsorption performance for QU. When the initial concentration of quercetin was 900 μg/ml, the adsorption capacity of UiO-66-BH-100 for QU was as high as 302.60 mg/g. Given that this material showed the best effect in QU adsorption, UiO-66-BH-100 was selected as the drug-loading material for QU in the subsequent experiments.

To elucidate the adsorption process of QU onto UiO-66-BH-100, the isothermal adsorption data were fitted using two well-established models: the Langmuir and Freundlich models. The fitting-derived parameters are summarized in Table 1. The results demonstrated that the Langmuir model (*R*^2^ = 0.9912) exhibited a superior fit compared to the Freundlich model (*R*^2^ = 0.9856), suggesting that the adsorption of QU onto UiO-66-BH-100 follows a monolayer adsorption mechanism (33).

**Table 1:** Adsorption models and corresponding statistical parameters.

**Sorbent**	**Langmuir isotherm equation**			**Freundlich isotherm equation**			**Pseudo-first-order model**			**Pseudo-second-order model**		
	*Q*_m_ **(mg/g)**	*K*_l_ **(L/mg)**	*R* ^2^	*K*_f_ **(mg/g)**	***m***	*R* ^2^	$Q_{\text{e}.\text{f}}^{\text{a}}$ **(mg/g)**	*K*_1_ **(min-1)**	*R* ^2^	$Q_{\text{e}.\text{f}}^{\text{b}}$ **(mg/g)**	*k*_2_ **(/min)**	*R* ^2^
UiO-66-BH-100	524.22	0.0013	0.9912	4.62	0.6064	0.9856	237.28	0.1284	0.7068	245.49	0.0013	0.8509

^a^Q_e, f_ (mg/g) is the calculated value of Q_e_ by PFO equation.

^b^Q_e, s_ (mg/g) is the calculated value of Q_e_ by PSO equation.

#### 3.1.2 Thermal alkaline hydrolysis conditions

Literature has demonstrated that variations in synthetic parameters between MOFs and alkaline solutions dictate the architectural features of the resulting materials (34). In the experiment, UiO-66-100 was mixed with the alkaline solution and reacted at room temperature to obtain the material UiO-66-RT-100. UiO-66 and the alkaline solution were heated separately, and then they were mixed and reacted to obtain UiO-66-SH-100. The experimental results showed that UiO-66-BH-100 obtained by mixing and heating UiO-66-100 with a KOH solution had the strongest adsorption effect on QU. This might be because the material obtained by mixing and heating had more suitable pores and active sites, which were beneficial to the adsorption of QU. Consequently, UiO-66-BH-100 was used as the sorbent material for QU for subsequent characterization and adsorption tests.

#### 3.1.3 Adsorption kinetics

An adsorption kinetic experiment was conducted to investigate the relationship between the equilibrium adsorption capacity of the material and the adsorption time. As shown in Figure 1B, UiO-66-BH-100 exhibited time-dependent adsorption enhancement for QU.

To deconvolute the QU adsorption mechanism on UiO-66-BH-100, the experimental kinetic data were subjected to non-linear regression analysis using PFO and PSO models. As summarized in Table 1, the PSO model demonstrated superior fitness to the PFO model (*R*^2^ = 0.8509 vs. *R*^2^ = 0.7068) and showed closer approximation to experimental values than PFO-derived values (237.28 vs. 302.60 mg/g). This statistical superiority confirms the dominance of chemisorption pathways involving potential electron donor-acceptor interactions between the UiO-66-BH-100 and QU (35).

**Figure 2:**
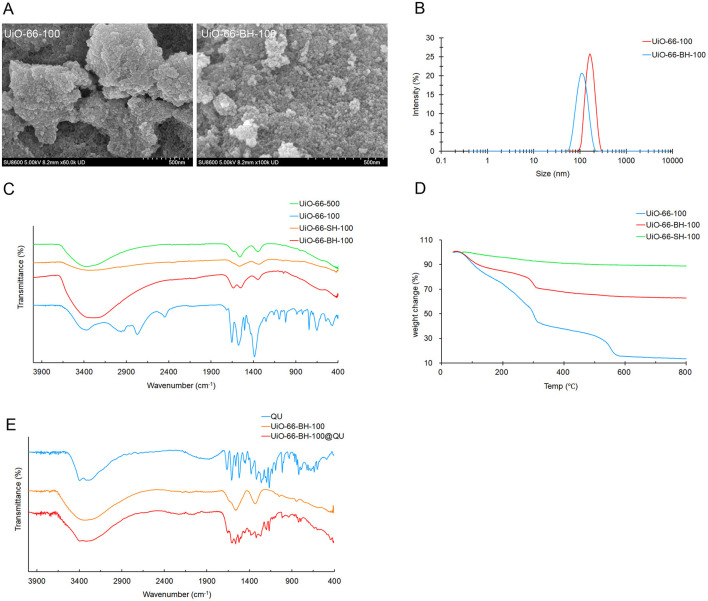
SEM images **(A)**, particle-size distribution **(B)**, IR profiles **(C, E)**, and TG profiles **(D)** of materials.

According to the literature (36, 37), the formation of hydrogen bonds can facilitate electron transfer. Our characterization result demonstrated that UiO-66-BH-100 was rich in hydroxyl groups. Based on this, we reasonably hypothesize that UiO-66-BH-100 can form intermolecular hydrogen bonds with quercetin (which is also rich in hydroxyl groups), and this, in turn, enables electron sharing between the two. To further verify the occurrence of electron sharing between UiO-66-BH-100 and quercetin (QU) during the adsorption process, we subjected QU, UiO-66-BH-100, and UiO-66-BH-100@QU to FTIR spectroscopy analysis, respectively (Figure 2E). It was found that the intensities of QU's characteristic peaks at 1,667 cm^−1^, 1,607 cm^−1^ (C=O stretching vibration), and 1,317 cm^−1^ (-C-OH- twist vibrations) (38) in UiO-66-BH-100@QU significantly decreased. This can be attributed to the electrons in QU being shared by UiO-66-BH-100, which leads to a decrease in the electron cloud density of the aforementioned groups and a subsequent weakening of the corresponding peak intensities.

### 3.2 Material characterization

As shown in Figure 2A, after the thermal alkaline hydrolysis treatment, the morphology of UiO-66-BH-100 changed. The particle size decreased significantly, and the surface became rough, which might be caused by the dissociation of ligands (26, 27). The rough surface provides space and binding sites for subsequent drug loading. The results of particle-size distribution are shown in Figure 2B. The particle-size distribution range of UiO-66-100 was approximately 160 nm, while that of UiO-66-BH-100, it was approximately 100 nm. The results indicate that the particle size of the material decreased after the treatment of mixing, heating, and alkaline hydrolysis.

The infrared (IR) spectrum of UiO-66-100 (Figure 2C) showed obvious characteristic absorption peaks of the terephthalic acid group (26, 39). After thermal alkaline hydrolysis treatment, a marked attenuation was observed in the out-of-plane bending modes of aromatic C-H oscillators at 666 and 743 cm^−1^ concomitant with diminished in-plane deformation vibrations from carboxylic -OH moieties at 1,400 cm^−1^. This distinct characteristic highlights the differences between UiO-66-BH-100 and conventional UiO-66 materials (40), which serves to confirm the dissociation of ligands in the former. It is worth noting that there was a strong absorption peak of the OH stretching vibration at ~3,305 cm^−1^ in UiO-66-BH-100. The existence of this characteristic peak strongly proved that UiO-66-BH-100 was rich in a large number of hydroxyl groups.

The results of TGA (Figure 2D) revealed the thermal decomposition behaviors of different materials. UiO-66-100 and UiO-66-BH-100 exhibited weight losses in the second and third stages, respectively. It is worth noting that both of them showed weight loss in the temperature range of 250–300 °C, and at this time, the weight loss might be attributed to the dehydration of surface hydroxyl groups (corresponding to -OH → *H*_2_O) (41). UiO-66-100 and UiO-66-BH-100 lost 14.0% and 7.6% of their weights, respectively, in the range of 250–300 °C. According to this weight loss data and the specific surface area measured by Brunauer–Emmett–Teller (BET), the surface hydroxyl density of UiO-66-BH-100 could be estimated to be approximately 1.7 × 10^19^ m^−2^. Obviously, in comparison, UiO-66-BH-500 showed almost no weight change in the temperature range of 250–300 °C, indicating that there were almost no hydroxyl groups on the surface of UiO-66-BH-500. The abundant hydroxyl groups in UiO-66-BH-100 might form more hydrogen bonds with QU, thus increasing the loading capacity of QU. UiO-66-SH-100 was heated up to 800 °C without obvious weight loss (the total weight loss was only 11.3%), demonstrating the super stability of UiO-66-SH-100. This confirmed that the operation of mixed thermal alkaline hydrolysis treatment after heating almost eliminated the terephthalic acid and surface hydroxyl groups in the material (which was consistent with the FTIR results), resulting in a stable material. At the same time, the elimination of the binding sites also limited the adsorption of the active ingredient QU by the material.

The results of the pore volume, surface area, and pore size of materials are shown in Table 2. The pore volume of UiO-66-100 was 0.18 cm^3^/g, and the Brunauer–Emmett–Teller (BET) surface area was 424.02 m^2^/g. Compared with UiO-66-500 (298.71 m^2^/g, 0.04 cm^3^/g), they were increased by 42 and 350%, respectively. The corresponding adsorption capacity of QU increased from 31.75 to 126.49 mg/g, and the adsorption capacity was positively correlated with the BET surface area. Compared with UiO-66-100, the BET surface area of UiO-66-BH-100 decreased to 292.45 m^2^/g (a decrease of 31%), but the pore volume increased to 0.29 cm^3^/g (an increase of 61%). Although the surface area was reduced, the adsorption capacity of UiO-66-BH-100 for QU significantly increased to 302.60 mg/g, far exceeding that of UiO-66-BH-500 (119.13 mg/g). This phenomenon might be attributed to the higher density of hydroxyl functionalities in UiO-66-BH-100, which contains more hydroxyl groups and could bind abundant QU through hydrogen bonds. Additionally, the expanded pore architecture also contributed to the enhancement of the adsorption capacity. When compared with the previously reported UiO-66 materials (40), UiO-66-BH-100 does not exhibit the most prominent specific surface area or porosity. However, benefiting from the ligand dissociation process, a considerable number of hydroxyl groups have been introduced into UiO-66-BH-100, which facilitates the loading of active components. On the other hand, the ligands obtained after thermal alkaline hydrolysis could be recovered and reused for the synthesis of MOF materials (34), thereby reducing raw material consumption.

**Table 2:** Surface area and porosity of the materials.

**Materials**	**Surface area (m^2^/g)**	**Pore volume (cm^3^/g)**	**Pore size (nm)**
UiO-66-100	424.02	0.18	3.63
UiO-66-BH-100	292.45	0.29	3.80
UiO-66-SH-100	160.28	0.09	3.05
UiO-66-500 (45)	298.71	0.04	5.08
UiO-66-BH-500 (45)	107.34	0.30	11.48

### 3.3 Drug payload and release

The drug payload capacity of UiO-66-BH-100 was quantified using a validated calibration curve derived from standardized analytical protocols. After calculation, the drug payload of QU by UiO-66-BH-100 was 20.92% ± 1.19%, which was significantly higher than that of QU by UiO-66-BH-500 (13.20% ± 0.07%).

**Figure 3:**
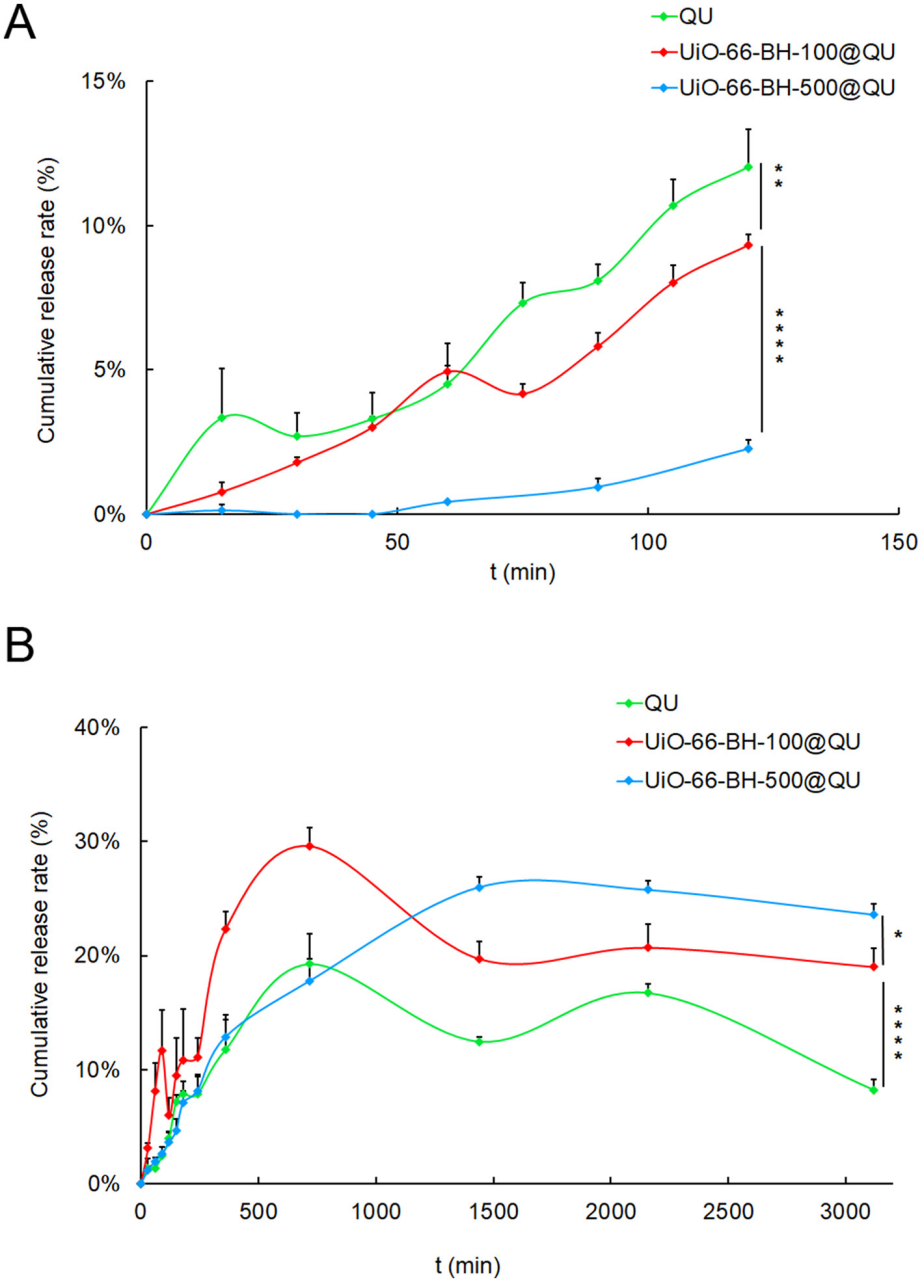
Release process of loaded QU in SGF **(A)** and SIF **(B)** conditions, *n* = 3. Statistical significance difference against UiO-66-BH-100@QU (*p < 0.05, **p < 0.01, ****p < 0.0001).

The pH value of the release medium has a significant impact on the release situation. To better simulate the real situation in the body, an acidic medium was used in the early stage of release, and an alkaline medium was used in the later stage of release. The release of QU in the drug-loaded system in the SGF and SIF is shown in Figure 3.

In SGF, QU was continuously released over time. At 120 min, the release amount reached 12.02%. The release behavior of QU in UiO-66-BH-100@QU was almost equivalent to that of pure QU. At 120 min, the release amount was 9.33%. However, the QU in UiO-66-BH-500@QU was hardly released in the SGF, and the release amount at 120 min was only 2.27%. In SIF, quercetin was continuously released. At 720 min, the release amount reached 19.25%. Subsequently, due to the poor stability of QU, its concentration fluctuated. The release behavior of QU in UiO-66-BH-100@QU was similar to that of pure QU, but the total release amount was higher than that of pure QU. At 720 min, the release amount reached 29.62%. The release behavior of QU in UiO-66-BH-500@QU in the SIF was different from the above two cases. The QU in it was continuously released, reaching a peak of 25.99% at 1,440 min, and then was almost maintained at this level. The above results indicated that UiO-66-BH-100 could maintain the release behavior and release amount of QU in SGF and could significantly improve the release behavior and release amount of QU in SIF.

### 3.4 Cytotoxicity assessment

**Figure 4:**
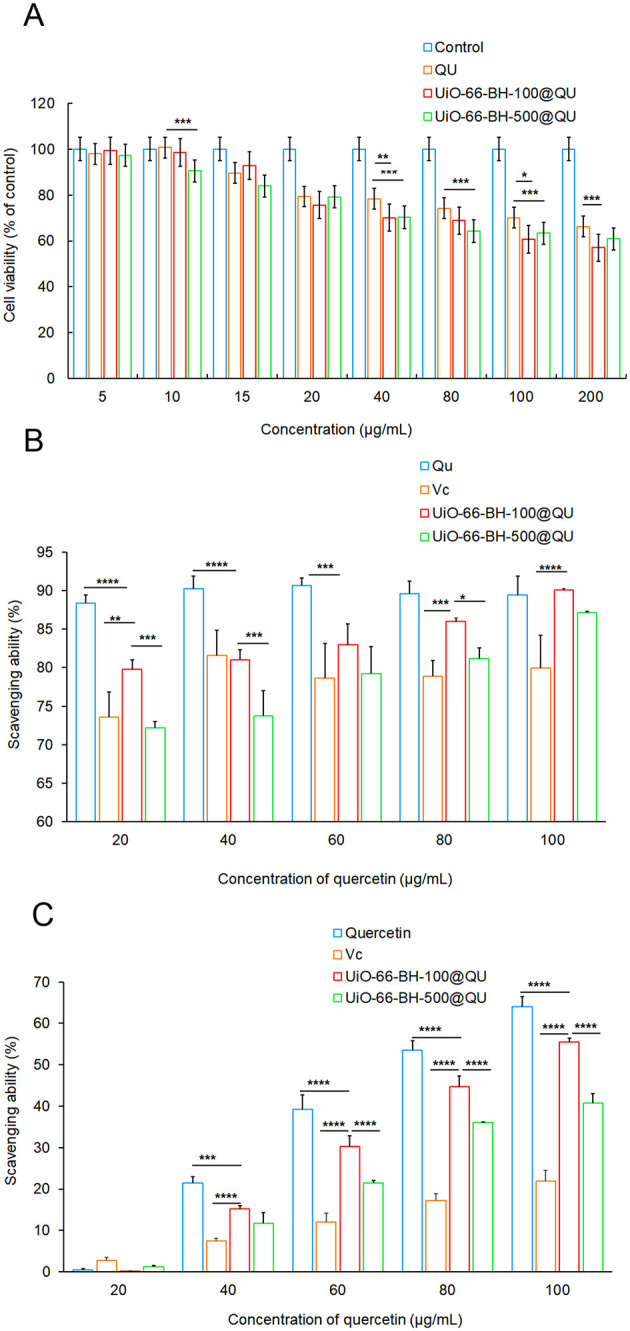
Cell viability value of RAW264.7 cell **(A)**, ABTS scavenging activity **(B)**, and DPPH scavenging activity **(C)** of encapsulated QU, *n* = 3. Statistical significance difference (*p < 0.05, **p < 0.01, ***p < 0.001, and ****p < 0.0001).

The cytotoxicity of two drug delivery systems with distinct particle sizes against RAW264.7 cells was evaluated using the MTT assay. As shown in Figure 4A, at QU concentrations of 5, 10, 15, 20, 40, 80, and 100 μg/ml, neither of the drug delivery systems exhibited significant inhibitory effects on the viability of RAW264.7 cells. These findings suggested that both drug delivery systems held considerable promise for safe pharmaceutical applications, which underscored their potential suitability for therapeutic use.

### 3.5 Antioxidant capacity tests

#### 3.5.1 ABTS radical scavenging ability

The results of the ABTS radical scavenging ability of the QU bulk drug and the drug delivery systems are shown in Figure 4B. The ABTS radical scavenging ability of the QU bulk drug increased with the increase in QU concentration. In contrast, although the ABTS radical scavenging ability of the two drug delivery systems decreased to some extent, they exhibited strong ABTS radical scavenging activity. Moreover, this scavenging ability gradually increased with the increase in the concentration of the drug delivery systems, showing a trend similar to that of QU. It is worth noting that, in terms of release behavior and release amount, UiO-66-BH-100@QU had a significant advantage over UiO-66-BH-500@QU. Based on this, among the drug delivery systems with the same QU content, UiO-66-BH-100@QU with a smaller particle size demonstrated a more potent ABTS radical scavenging ability.

#### 3.5.2 DPPH radical scavenging ability

As shown in Figure 4C, the DPPH radical scavenging assay results revealed that varying concentrations of the QU did not exhibit any significant differences in their DPPH radical scavenging capabilities. When the drug-loading material was loaded with QU, the DPPH radical scavenging ability was lower than that of QU at the same concentration. Nevertheless, due to the higher QU release rate of the UiO-66-BH-100@QU compared to the UiO-66-BH-500@QU, the UiO-66-BH-100@QU exhibited a more excellent DPPH radical scavenging ability than the UiO-66-BH-500@QU under the same concentration condition. Based on the experimental results of the ABTS and DPPH assays, the UiO-66-BH-100@QU drug delivery system exhibited superior free radical scavenging activity compared to those reported in the literature (42). Meanwhile, Vc was used as a standard antioxidant to compare the antioxidant activity results of the aforementioned drug delivery systems. As shown in Figures 4B, C, the ability of Vc to scavenge ABTS and DPPH free radicals at the same concentration was significantly lower than that of QU, which is consistent with previous literature reports (43). Based on the combined results of the ABTS and DPPH scavenging assays (Figures 4B, C), it can be concluded that the UiO-66-BH-100@QU exhibited superior antioxidant effects to Vc at the same concentration, and its ABTS and DPPH scavenging capacities were comparable to those of free QU. Notably, the samples were dissolved in an absolute ethanol system for the antioxidant activity evaluation. Given that the release behavior of QU from the UiO-66-BH-100@QU system in a simulated gastrointestinal fluid environment was significantly better than that of free QU (29.62 vs. 19.25%), we reasonably speculate that the UiO-66-BH-100@QU drug delivery system may possess superior antioxidant capacity to free QU *in vivo*.

### 3.6 *In vitro* anti-inflammatory study

**Figure 5:**
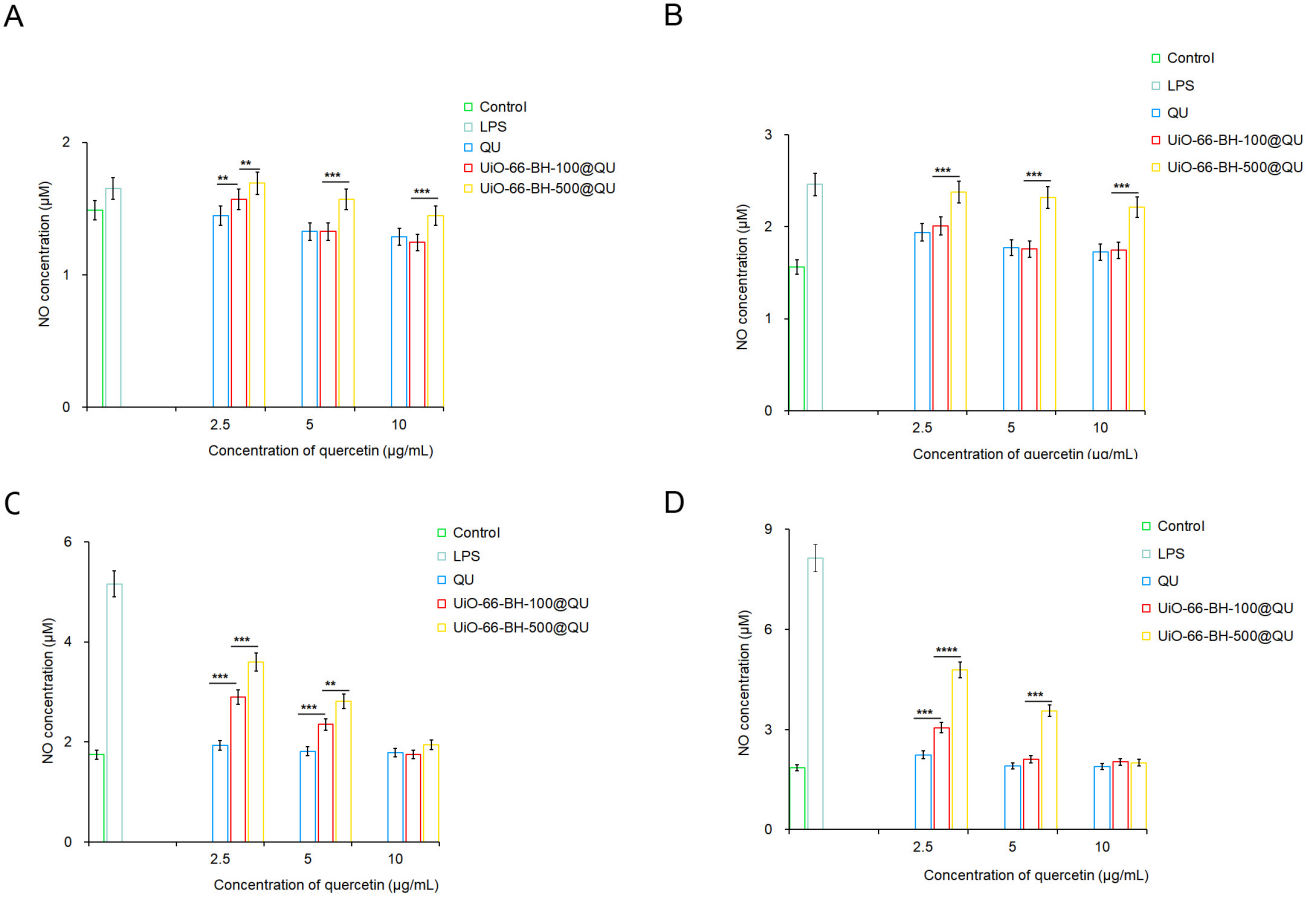
Effects of encapsulated QU on NO secretion by RAW264.7 cells at 6 **(A)**, 12 **(B)**, 18 **(C)**, and 24 h **(D)**, *n* = 3. Statistical significance difference against UiO-66-BH-100@QU (**p < 0.01, ***p < 0.001, and ****p < 0.0001).

As a well-known pro-inflammatory agent, LPS activates RAW264.7 cells, triggering substantial NO secretion and inflammation. To assess the anti-inflammatory effects of quercetin-loaded materials with different particle sizes, we established an LPS-induced RAW264.7 cell injury model and measured NO levels in the culture supernatant. Figure 5 shows the impacts of QU and the drug delivery systems on NO secretion by RAW264.7 cells.

NO secretion by cells in the LPS group continuously increased at 6, 12, 18, and 24 h, confirming the successful establishment of the cell injury model. The QU treatment groups inhibited LPS-induced NO secretion across different time points and concentrations, which is consistent with previous studies demonstrating QU's anti-inflammatory properties. QU likely reduces NO secretion by suppressing relevant inflammatory signaling pathways and decreasing the expression or activity of key enzymes involved in NO synthesis (36). Notably, due to QU's poor water solubility, oral administration may not achieve the same potent anti-inflammatory effects observed in this cell-based experiment.

Despite containing the same amount of QU, UiO-66-BH-100@QU and UiO-66-BH-500@QU exhibited distinct efficacies in inhibiting NO production across different time points. Notably, UiO-66-BH-100@QU demonstrated a significantly superior capacity to suppress NO generation compared to UiO-66-BH-500@QU. At 6, 12, and 24 h, when QU concentration was 5 μM, UiO-66-BH-100@QU showed nearly equivalent NO-inhibiting performance to the QU bulk drug. In contrast, UiO-66-BH-500@QU required a higher concentration (10 μM at 18 and 24 h) to achieve a comparable level of NO suppression. These findings highlight that UiO-66-BH-100@QU could effectively facilitate QU's NO-inhibiting activity at lower concentrations, matching the efficacy of the bulk QU drug. UiO-66-BH-100@QU exhibited the ability to inhibit the release of NO that was superior to previous drug delivery systems (31, 44).

This study systematically revealed the significant advantages of the UiO-66-BH-100 drug delivery system in the field of QU delivery through multidimensional experiments. The small particle size and ligand detachment gave UiO-66-BH-100 a larger specific surface area, abundant hydroxyl groups, and higher surface energy. These not only provided more adsorption sites for QU but also enhanced the interaction between the drug-loading material and QU molecules, thereby increasing the drug loading capacity. Based on the verification of the Langmuir adsorption model and the PSO kinetic equation, it was clarified that the adsorption of QU by this material is mainly monolayer chemical adsorption. The cytotoxicity experiment confirms its good biocompatibility. During the drug release stage, due to the large surface contact area, QU in UiO-66-BH-100@QU could diffuse and be released more quickly, thus exhibiting antioxidant performance comparable to that of QU to a certain extent. Meanwhile, in the LPS-induced inflammatory model, UiO-66-BH-100@QU could achieve the same NO inhibition effect as bulk QU at lower concentrations, confirming its potential in anti-inflammatory applications. Given that the developed UiO-66-BH-100 exhibits excellent QU loading capacity, controls the release of QU in SIF, and simultaneously protects QU from degradation, these advantages of the release system are crucial for overcoming the issues of low solubility and poor bioavailability of QU.

## 4 Conclusion

Overall, the UiO-66-BH-100@QU drug-loaded system, with its unique structural characteristics and particle size advantages, not only effectively improves the release behavior of QU but also enhances antioxidant and anti-inflammatory activities through precise control of drug release. It provides an innovative solution for the development of natural active ingredients such as QU, which have poor water solubility and low bioavailability. Through the optimization of carrier materials, this study offers insights for improving QU “existence form, absorption efficiency, and functional performance” in food and expands the application prospects of MOF carrier materials in the field of delivering natural active ingredients in food systems. In the future, in the food field, the biological safety of carrier materials can be further evaluated through simulated gastrointestinal digestion experiments. On this basis, the compatibility or adaptability between drug-loaded systems and food matrices can be explored to facilitate the industrialization process of QU-based functional foods. By appropriately dispersing the release system developed in this study in liquid foods (e.g., beverages and dairy products), solid foods (e.g., baked goods), or dietary supplements, it helps enhance the stability of QU in the gastrointestinal environment and improve the absorption efficiency of QU in the human body, thereby providing a new approach for the development of functional health foods with anti-inflammatory and antioxidant effects.

## Data Availability

The original contributions presented in the study are included in the article/supplementary material, further inquiries can be directed to the corresponding authors.
